# Essential oil supplementation improves insulin sensitivity and modulates the plasma metabolome of hyperinsulinemic horses

**DOI:** 10.3389/fvets.2024.1444581

**Published:** 2024-12-02

**Authors:** Caroline M. M. Loos, Shuang Zhao, Liang Li, Janet Li, Wei Han, Eric S. Vanzant, Kyle R. McLeod

**Affiliations:** ^1^Department of Animal and Food Sciences, University of Kentucky, Lexington, KY, United States; ^2^The Metabolomics Innovation Centre and Chemistry Department, University of Alberta, Edmonton, AB, Canada

**Keywords:** essential oils, insulin dysregulation, metabolomics, horse, biomarker

## Abstract

The objective of this study was to investigate the effect of essential oil (EO) supplementation on insulin sensitivity (IS) and the plasma metabolome in insulin dysregulated (ID) horses. Horses were blocked by degree of IS and assigned randomly to treatment: oral daily bolus (50 mL) of either a plant derived EO supplement or carrier (CON). Mares were housed in dry lots with *ad libitum* access to grass hay and supplemented individually twice daily with a concentrate to meet nutrient requirements for mature horses. Before and after 6 wks of treatment, mares underwent a combined glucose-insulin tolerance test (CGIT) and an oral sugar test (OST) on separate days. Global metabolome analysis was conducted on plasma samples before and after treatment. Although treatment did not affect (*p* > 0.4) AUC or glucose clearance during CGIT, there was a treatment*covariate interaction (*p* ≤ 0.08) for insulin concentrations at 75 min (INS75) and positive phase time (PT) with EO decreasing both INS75 (*p* ≤ 0.002) and PT (*p* = 0.05) in horses with more severe initial degree of ID. Similarly, EO treatment reduced (*p* ≤ 0.006) insulinemic response to the OST in horses exhibiting higher pre-treatment responses (treatment*covariate, *p* = 0.004). There were 702 metabolites identified that were uniquely changed with EO treatment. Pathway analysis and biomarkers showed EO-mediated changes in amino acid, linoleic acid, mesaconic acid, TCA-cyle intermediates and bile acid metabolism. The directional changes in these pathways or biomarkers are consistent with changes in inulin sensitivity in other models. These data show that EO shifted the plasma metabolome and improved insulin sensitivity in horses.

## Introduction

Insulin dysregulation (ID), characterized by hyperinsulinemia and an excessive response to an oral or intravenous glucose challenge, is a core component of the pathophysiology of equine metabolic syndrome (EMS) (1–3). Symptoms associated with EMS have considerable impact on performance and general well-being of the horse, with laminitis being one of the most common and often detrimental clinical conditions seen in equine veterinary practice. How ID may contribute to the onset of laminitis has yet to be fully elucidated, but proposed mechanisms include hyperinsulinemia-induced hypoperfusion, haemodynamic changes and increased inflammation of the lamellar tissues (4). While significant progress has been made in the ability to manage laminitic horses, the overall prognosis of affected animals remains poor, with a high risk of lifelong recurrent health problems. Management strategies to mitigate ID are therefore essential not only in the treatment but more importantly, in the prevention of laminitis in predisposed animals. Albeit significant research efforts, few science-based treatments currently exist that effectively and consistently improve ID.

The multifactorial pathology of ID makes proper diagnosis and consequently the identification of a treatment target a particularly difficult task. The term “ID” is an all-encompassing term to describe any abnormality in insulin metabolism manifesting in hyperinsulinemia. However, the underlying cause of the dysregulation is often unclear and is known to involve a multitude of organs and systems, including pancreas, liver, adipose and muscle tissue as well as the immune system. To gain a more holistic perspective of the cross-talk and synergistic effect of different systems in clinical pathologies, “omics” studies have been proven extremely useful to capture general shifts in major physiological pathways. Previous metabolomics work for example has illustrated differences between non-ID and ID individuals, including horses (5). The use of these techniques could be particularly useful in evaluating treatment effects on a complex pathology such as ID.

Plant-derived extracts, such as essential oils, are known to have health benefits mediated by specific physiological effects of their biologically active compounds, including terpenes and terpenoids (6). Many essential oils are known to have antioxidant, antibacterial, antifungal and anti-inflammatory properties (7). Others, such as garlic, ginger and lavender have been suggested to alleviate metabolic problems by modulating lipid metabolism and oxidative stress (8–10). Moreover, research in humans and rodents have illustrated the insulin sensitizing effects of certain essential oils (10, 11). Suggested mechanisms by which certain essential oils might improve glucose metabolism include increased glucose transporter expression and upregulation glycolysis related enzymes and downregulation of gluconeogenesis related enzymes (12). Although some research has investigated the potential use of essential oils as nutraceuticals in horses (13–15), their effects on insulin dysregulation (ID) remain unexamined.

Therefore, the objective of the study was to determine the effects of a commercial essential oil blend on hyperinsulinemia and insulin sensitivity in horses diagnosed with ID and evaluate the effect of essential oil treatment on the plasma metabolome. It was hypothesized that essential oil supplementation would improve insulinemic responses to an oral and intravenous glycemic challenge and cause a differential shift in the plasma metabolome.

## Materials and methods

### Animals and housing

Twenty mature, insulin dysregulated (ID) mares (mean age 15.6 ± 3.0 years) of mixed breed were selected from the University of Kentucky, Department of Veterinary Science research herd. ID horses were identified based on their insulin status as determined by an oral sugar test (OST) as previously described (16). Briefly, two blood samples were collected via jugular venipuncture before and 60 min after administration of 0.15 mL/kg body weight (BW) of Karo light corn syrup. Based on criteria published in 2020 by the Equine Endocrinology Group (EEG), 20 insulin dysregulated (ID) were identified for the study, with ID defined as plasma insulin concentrations >20 μIU/mL and > 45 μIU/mL at baseline and 60 min post OST, respectively (17). Based on basal ACTH concentrations and evaluation of clinical symptoms, none of the horses in the study were considered to have PPID (Pituitary Pars Intermedia Dysfunction). While some of the horses has a history of laminitis, none exhibited signs of laminitis at onset or during the study.

To minimize the impact of changes in housing and diet on the parameters of interest, horses were kept in their familiar groups and housing conditions and received the same hay and concentrate feed that they had been maintained on for at least 1 year. Horses were housed in group in 2 adjacent outdoor dry lot pens (*n* = 10 per pen) with free access to grass hay round bales (Table 1) and a salt and mineral block. Assuming a min. of 1.5% BW in hay intake, each horse was individually fed 0.16% of BW of a commercial concentrate (StaminOats, Hallway Feeds, Lexington, KY, USA, Table 1) twice daily, so that the total diet provided 110% of daily DE requirements. This amount of feed also provided sufficient starch (0.6 g/kg BW/meal) to maintain horses in their ID status. Additionally, a custom-made vitamin-mineral premix pellet (Cavalor Feeds and Supplements, Deinze, Belgium) was added to the daily meals at the manufacturer’s recommended level of 100 g/day. This dietary regimen was designed to meet or exceed all nutrient needs for mature, idle horses with “average” maintenance requirements (18). There were no grain refusals recorded throughout the entire study. Each horse was adjusted to individual feeding procedures for at least 2 weeks prior to the first sample collections. Horses were weighed on an electronic scale weekly and condition score (BCS) was assessed at the beginning and end of the study. BCS was determined on a 1–9 scale (19) by 2 independent scorers, blinded to the experimental design.

**Table 1:** Nutrient composition of the daily ration on dry matter basis.

Nutrient	Concentrate	Grass hay
	% of DM	
DE (Mcal/kg)*	3.35	2.12
Crude protein	16.8	14.1
Acid detergent fiber	8.8	39.2
Neutral detergent fiber	22.3	60.3
Water-soluble carbohydrates	8.7	7.1
Ethanol-soluble carbohydrates	7.1	4.2
Starch	34.7	1.1
Non-fiber Carbohydrates	51.3	16.5
Calcium	0.98	0.63
Phosphorus	1.15	0.48
Magnesium	0.26	0.28
Potassium	1.12	2.57
Sodium	0.26	0.03
	PPM	
Iron	300	365
Zinc	189	27
Copper	50	8
Manganese	148	81
Molybdenum	3.0	2.0

*DE calculated value (70).

### Experimental procedures

In a randomized complete block design, horses were studied before (“pre” supplementation) and after (“post” supplementation) 6 weeks of treatment. In the “pre” sample period, all horses underwent a combined glucose insulin tolerance test (CGIT), a high starch feed challenge and an OST on 3 separate days with 1 recovery day between each sample day. Based on the CGIT results, horses were then blocked by degree of insulin sensitivity and randomly assigned (Table 2) to receive either the placebo (*n* = 10) or essential oil supplement (*n* = 10) such that treatment groups were balanced for insulin status. After the initial sample collection period, horses received an oral daily bolus of either a commercial plant-derived essential oil supplement (“EO,” *n* = 10, Cavalor LaminAid, Cavalor Feeds and Supplements, Drongen, Belgium) or the carrier oil (placebo, *n* = 10, Cavalor Feeds and Supplements, Drongen, Belgium) at the manufacturer recommended dose of 50 mL/day. The supplement was composed of a proprietary blend of 12 different essential oils (including *Eucalyptus globulus*, *Allium sativum* and *Betula alba*) with olive oil as a carrier. The composition of the placebo product was identical to supplement but without the active ingredients (i.e., essential oils). Treatments were given with an oral dosing syringe every afternoon, once horses had consumed their afternoon concentrate meals. After 6 weeks of supplementation, all 3 sample procedures were repeated in the “post” sample period.

**Table 2:** Phenotypic measures and insulin status for placebo and essential oil groups prior to the start of treatment.

Phenotypic measure	Placebo	Essential oils	*p*-values
Insulin OST 0 min (μIU/mL)	26.9 ± 4.04	33.0 ± 4.26	0.3
Insulin OST 60 min (μIU/mL)	95.3 ± 13.22	90.1 ± 13.22	0.8
CGIT glucose AUC (mg/dL/min)	2432.7 ± 353.65	2745.1 ± 353.65	0.5
Body weight (kg)	548.7 ± 18.23	590.0 ± 18.23	0.13
Body condition score	7.6 ± 0.27	7.7 ± 0.27	0.7
Age (years)*	14.3 ± 1.25	16.8 ± 1.36	0.2

CGIT, combined glucose insulin tolerance test; AUC, area under the curve; OST, oral sugar test. *n* = 10 placebo and *n* = 10 essential oils. *Age was not known for all horses due to limited history on horse donations (*n* = 6 known for placebo, *n* = 7 known for essential oils group). Data are presented as least square means ± standard error of the mean.

The day before each sample period each horse was weighed and an area over the jugular veins was clipped. Horses had free access to hay overnight, but morning grain meals were withheld on sample days. Each horse was placed into their individual holding pen 2 h prior to initiation of any sample procedures and had access to water at all times during sampling. Immediately following completion of each procedure, horses were fed their allotted concentrate meal and released into the dry lot pens. Blood samples were collected into heparinized vacutainer tubes at predetermined times for each procedure (see below). All blood samples were stored on ice and plasma harvested after centrifugation (1,500 × *g* for 10 min) when testing procedures were completed. Plasma samples were stored at −20°C until further analysis.

#### CGIT procedures

Before and after 6 weeks of supplementation, insulin sensitivity was assessed from a CGIT administered as previously described (20). Briefly, intravenous catheters were aseptically placed in the jugular vein after which horses were allowed to recover for approximately 30 min before starting the procedures. Two baseline blood samples (10 mL) were collected at −15 and 0 min and blood glucose levels measured immediately using a handheld glucometer, after which horses were rapidly (<1 min) infused intravenously with a bolus of sterile 50% dextrose solution that provided 150 mg/kg BW. Immediately following, a sterile intravenous insulin injection was given at a rate of 0.1 U/kg BW (diluted in approximately 3 mL of isotonic saline solution). Additional blood samples (10 mL) were collected 1, 5, 15, 35, 45, 60, 75, 90, 105, 120, 135 and 150 min after the insulin injection. To monitor glycemia, blood glucose concentrations were determined from each collected sample throughout the procedure using a handheld glucometer. No horses showed concerning signs of hypoglycemia and no emergency dextrose administration was needed during any of the procedures.

#### Feed challenge procedures

In further assessment of changes in insulin and glucose dynamics following essential oil supplementation, each horse was subjected to a feeding challenge before after 6 weeks of treatment. On the morning of each test, intravenous catheters were aseptically placed in the jugular vein after which horses were allowed to recover for approximately 30 min before starting the procedures. Two baseline blood samples were collected by jugular venipuncture at −30 and 0 min after which each horse received an amount of whole oats (52.8% starch on DM basis) that provided 1 g starch/ kg BW. This amount of cereal grain starch has previously shown to elicit a glycemic and insulinemic response in horses (21). Additional blood samples were collected by jugular venipuncture 60, 120, 180, 240 and 300 min after the meal was administered. Time to completion of the meal and any leftover grain was recorded. All horses consumed their meals in a timely manner and no leftovers were recorded.

All procedures were approved by the Institutional Animal Care and Use Committee at the University of Kentucky.

### Sample analyses

#### Plasma glucose and insulin analyses

Whole blood glucose concentrations during the CGIT procedure were measured immediately after collection using a handheld glucometer (Accu-Chek Aviva, Roche Diagnostic, Indianapolis, IN, USA). Plasma glucose concentrations from samples collected during the feed challenge and OST were determined enzymatically using an automated analyzer (YSI 2700 Select Analyzer, YSI Inc., Yellow Spring, OH, USA). Plasma insulin levels were assayed for CGIT at *t* = 75 min and all samples collected for the feed challenged and OST using a commercially available radioimmunoassay kit (PI-12 K, Millipore Sigma, Burlington, MA, USA) validated for horses as previously described (22). All plasma samples were run in duplicate. Average intra and inter-assay variation for glucose was 0.3 and 3.4%, respectively. Average intra and inter-assay variation for insulin was 7.6 and 7.4%, respectively.

#### Analysis of the CGIT results

Analysis of the CGIT results was conducted as previously described (23). Briefly, “positive phase” is defined as the period of the CGIT procedure during which glucose concentrations are elevated above baseline (*t* = 0 min) concentrations, not including glucose recovery (i.e., period where glucose concentrations rise above baseline after reaching nadir concentrations). The positive phase duration (defined as “time in positive phase”) was calculated as the time (min.) between the start of the CGIT (*t* = 0 min) until the timepoint of lowest measured glucose concentration in the positive phase (defined as “lowest positive phase glucose concentrations”). Time until nadir was calculated as the time between the start of the CGIT (*t* = 0) until the time of lowest measured glucose concentration during the entire CGIT procedure. Positive phase glucose clearance rate was calculated by dividing the difference between peak and lowest positive phase glucose concentrations by the difference in time between positive phase duration and time to reach peak glucose concentrations. Delta *t* = 45 min was calculated as the difference between glucose concentrations at 45 min and baseline (*t* = 0) glucose concentrations. Horses were considered insulin dysregulated when the positive phase duration >45 min or if plasma insulin concentrations were > 20 μIU/mL at 75 min (20). Area under the glucose response curve (AUC) was evaluated for the positive phase (i.e., area under the positive response curve until baseline glucose concentrations were reached). The negative phase (i.e., glucose levels below baseline) or recovery phase (i.e., glucose concentrations above baseline near the end of the CGIT) were not considered in the final analysis as it would be impossible to distinguish between glucose clearance and hepatic glucose production at this point.

#### Plasma metabolome analyses

Global metabolome analysis was conducted on plasma samples using the Chemical Isotope Labeling LC–MS (CIL LC–MS) approach as previously reported (24). Briefly, the whole metabolome was analyzed by targeting four submetabolomes: amine/phenol-, carboxyl-, hydroxyl- and carbonyl-submetabolome. For each submetabolome, samples were derivatized with a pair of isotopic labeling reagents (i.e., 12C-/13C-reagents) prior to LC–MS analysis. Individual samples were labeled with 12C-reagents and a pooled sample, which was generated by mixing an aliquot from each individual sample, was labeled with 13C-reagents. After labeling, the 12C-labeled individual sample was mixed with the same amount of the 13C-labeled pool, followed by LC–MS analysis. In the mass spectra, each metabolite was detected as a peak pair, i.e., the light peak from the 12C-labeled individual sample and the heavy peak from the 13C-labeled pool. The peak intensity ratio between 12C-peak and 13C-peak represents the relative quantification result for a specific metabolite in an individual sample.

In this study, proteins from the samples were first precipitated by adding three times of pre-cold methanol into the individual samples. After incubation and centrifugation, the supernatants were taken and dried down, then re-dissolved in LC–MS grade water. Chemical isotope labeling of each sample was carried out by following the standard operating procedures (SOPs) provided in the labeling kits (Nova Medical Testing Inc., Product Numbers: NMT-4101-KT, NMT-4123-KT, NMT-4145-KT, and NMT-4167-KT). For each labeling experiment, an aliquot of 25 μL of sample was used. The amine−/phenol metabolites were labeled using dansylation reaction. The carboxyl metabolites were labeled using DMPA bromide. The hydroxyl metabolites were labeled using base-activated dansylation reaction. The carbonyl metabolites were labeled using dansylhydrazine.

After labeling, the 12C-labeled individual sample was mixed with 13C-labeled pool sample in equal volume. The mixture was injected into LC–MS for analysis. All LC–MS analysis were carried out on using Agilent 1,290 LC linked to Bruker Impact II QTOF Mass Spectrometer. The column used was Agilent eclipse plus reversed-phase C18 column (150 × 2.1 mm, 1.8 μm particle size) and the column oven temperature was 40°C. Mobile phase A was 0.1% (v/v) formic acid in water and mobile phase B was 0.1% (v/v) formic acid in acetonitrile. The gradient setting was: *t* = 0 min, 25% B; *t* = 10 min, 99% B; *t* = 15 min, 99% B; *t* = 15.1 min, 25% B; *t* = 18 min, 25% B. The flow rate was 400 μL/min. Mass spectral acquisition rate was 1 Hz, with an m/z range from 220 to 1,000.

### Data analysis

#### Metabolomics data processing and analysis

After data acquisition, raw LC–MS data were uploaded to IsoMS Pro 1.2.10 (Nova Medical Testing Inc.) for data processing and metabolite identification. 12C-/13C-peak pairs in each sample were first extracted and peak intensity ratio was calculated for each peak pair. Peak pairs that were not presented in at least 80.0% of samples in any group were filtered out. Metabolite identification was carried out using a three-tiered approach against NovaMT Metabolite Databases 2.0 (Nova Medical Testing Inc.). In tier 1, peak pairs were searched against a labeled metabolite library (CIL Library) based on accurate mass and retention time, considering as positive identification. In tier 2, the remaining peak pairs were searched against a linked identity library (LI Library), which includes over 9,000 pathway-related metabolites, providing high-confidence putative identification results based on accurate mass and predicted retention time matches. In tier 3, any remaining peak pairs were searched, based on accurate mass match, against the MyCompoundID (MCID) library^1^ (20) composed of 8,021 known human endogenous metabolites (zero-reaction library), their predicted metabolic products from one metabolic reaction (375,809 compounds) (one-reaction library) and two metabolic reactions (10,583,901 compounds) (two-reaction library). In this study, global metabolomics analysis detected 10,281 metabolites by using the CIL LC–MS approach. Among them, 566 metabolites were identified in Tier 1, 1939 in Tier 2 and 6,312 in Tier 3.

All detected metabolites were submitted to IsoMS Pro and MetaboAnalyst 5.0^2^ for further analysis. Multivariate analyses, including principal component analysis (PCA) and partial least squares discriminant analysis (PLS-DA), were used to visualize the metabolome profiles. To identify significantly up-regulated and down-regulated metabolites, volcano plot as univariate analysis was employed by using *p*-value <0.05, fold change >1.2 or < 0.83 as the cut-off. Heatmap as cluster analysis was used to present the clustering of samples. Based on initial PLS-DA analysis, one horse was identified as an outlier in the post-essential oil group as it fell outside of all confidence ellipses and deviated substantially from its treatment cohort along the PC1 by greater than a score of 60. Thus, both pre and post supplementation data for this horse were removed from further analysis.

#### Statistical analysis of glucose and insulin responses

Data analysis was performed using mixed procedures in SAS 9.4 statistical software (SAS Institute, Cary, NC, USA) with significance considered at *p* ≤ 0.05 and trends considered when 0.05 < *p* ≤ 0.10. Variables measured over time (feed challenge) were analyzed using repeated measures two-way ANOVA with appropriate variance–covariance structures chosen for each variable based on lowest AICC fit statistics, and the kr2 adjustment for degrees of freedom. Treatment, time, and their interactions were considered fixed effects. Additionally, pre-treatment values were included as a covariate in the model (i.e., “pre”). Block (i.e., horse pen) was considered a random factor in the model. Time was considered the repeated measure with horse a as the subject. All other variables were analyzed using a one-way ANOVA with treatment the as fixed effects and block as a random factor. Pre-treatment values were included as a covariate in the model (i.e., “pre”). Where the treatment and covariate interaction was significant, the treatment effect was evaluated at three pre-determined levels of the covariate (i.e., minimum, average and maximum values of the overlapping data points between the treatments) using an independent slope model. If the covariate by treatment interaction was not significant, a common slope model was used to assess treatment effects at one level of the covariate (i.e., the average level of the covariate).

Studentized residuals for all response variables were evaluated graphically (using Q/Q plots) and with the SAS univariate procedures to ensure adherence to normality assumptions. Due to non-normal distribution of the studentized residuals, glucose and insulin data from the feeding challenge were log-transformed for analysis. Outliers were identified from studentized residuals >3 SD from the mean and were removed from the data set where appropriate. All data are presented as least square means and standard error of the mean unless otherwise noted.

## Results

### Body weight and BCS

Body weight and BCS were not affected by treatment (data not shown, *p* ≥ 0.7). After 6 weeks of supplementation average body weight was 571.6 ± 3.14 and 570.8 ± 3.14 kg for the placebo and essential oil group, respectively (*p* = 0.7). Average BCS was 7.3 ± 0.20 for the placebo and 7.3 ± 0.20 for the essential oil group at the end of the supplementation period (*p* = 0.8).

### Combined glucose insulin tolerance test

**Figure 1:**
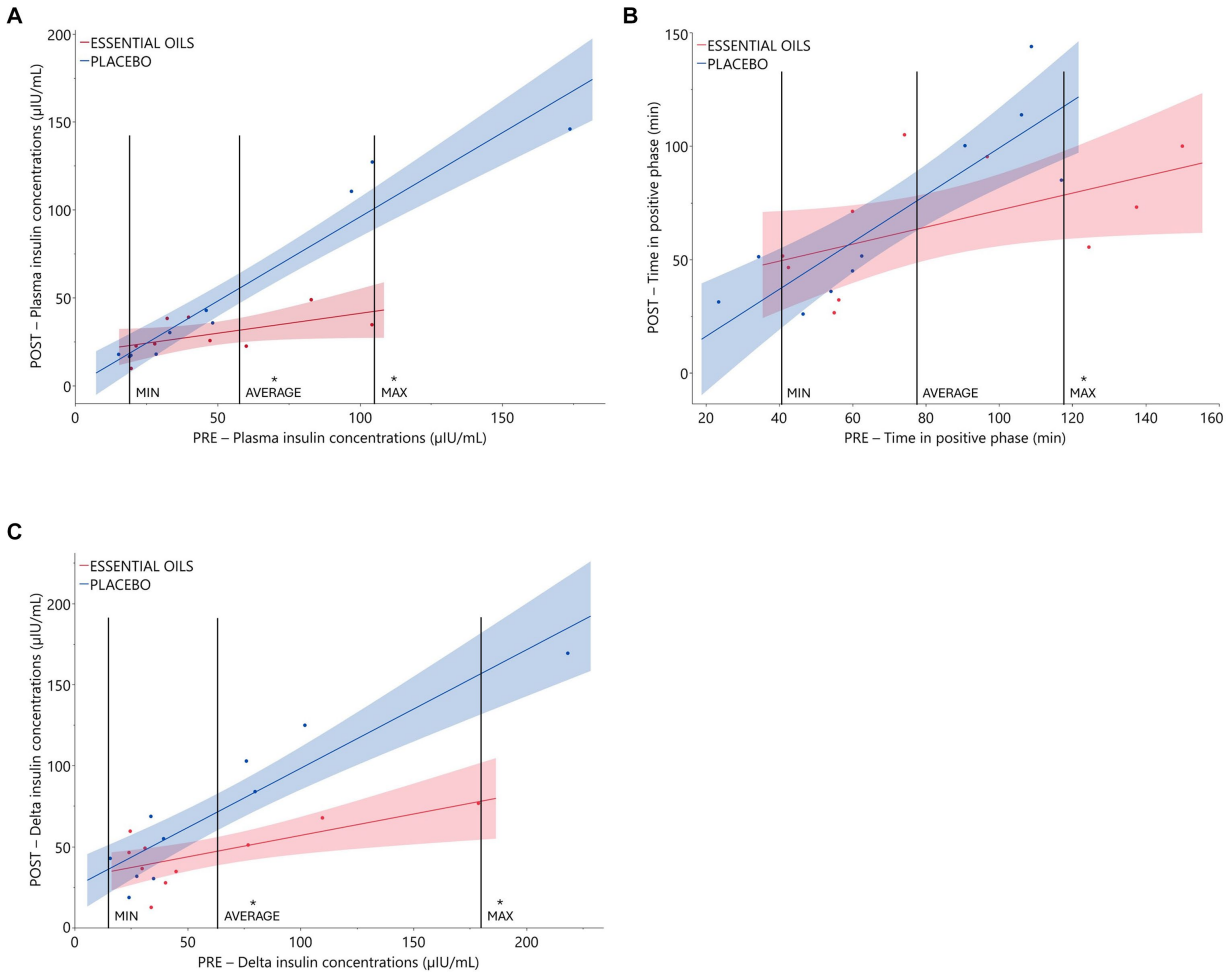
Relationship between initial insulin status and treatment effect on CGIT and OST parameters. Plasma glucose and insulin responses during CGIT and OST. Data presented as regression of pre (covariate) vs. post measurements using an independent slope model with 90% CI. Treatment effects were evaluated at different levels of the covariate (i.e., minimum, average and maximum levels). **(A)** Pre and post insulin concentrations (μIU/mL) at 75 min during CGIT (treatment*covariate interaction *p* = 0.0008, placebo *n* = 10, essential oils *n* = 9). *Indicates treatment differences at the respective level of the covariate (*p* ≤ 0.002); **(B)**: time in positive phase (i.e., time for glucose to return to basal concentrations) during CGIT (treatment*covariate interaction *p* = 0.04, placebo *n* = 10, essential oils *n* = 10). *Indicates treatment differences at the respective level of the covariate (*p* = 0.03). **(C)**: insulin response (i.e., delta: 60 min – 0 min insulin concentrations) to oral sugar administration (treatment*covariate interaction *p* = 0.008, placebo *n* = 10, essential oils *n* = 10). *Indicates treatment differences at the respective level of the covariate (*p* ≤ 0.006).

Six weeks of essential oil treatment reduced time in the positive phase (treatment*covariate *p* = 0.04) and insulin concentrations at 75 min (treatment*covariate *p* = 0.0008), two markers of insulin sensitivity, in horses with severe initially degree of insulin resistance (Table 3 and Figure 1). Insulin concentrations at 75 min were reduced after 6 weeks of EO treatment for ID horses with “pre” insulin concentrations ≥55.8 μIU/mL. Horses with an average insulin concentration of 55.8 μIU/mL at the start of the study (i.e., the average level of the covariate) had plasma insulin concentrations of 53.5 ± 4.04 and 31.1 ± 4.41 μIU/mL post supplementation in the placebo and EO group, respectively (*p* = 0.002, Figure 1A, Table 3). Horses with an average insulin concentration of 104 μIU/mL at the start of the study (i.e., the maximum level of the covariate) had plasma insulin concentrations of 99.74 ± 5.53 and 41.97 ± 9.63 μIU/mL after supplementation in the placebo and EO group, respectively (*p* = 0.0001, Figure 1A). Additionally, horses with a positive phase time ≥ 117 min at the start of the study (i.e., the maximum level of the covariate) had a positive phase time of 116.8 ± 13.23 and 78.2 ± 9.74.25 min post supplementation in the placebo and EO group, respectively (*p* = 0.03, Figure 1B, Table 3). EO supplementation did not affect any other CGIT parameters (*p* ≥ 0.2, Table 3).

**Table 3:** Effect of placebo or essential oils on CGIT and OST parameters after 6 weeks of supplementation.

			*p*-values		
	Placebo	Essential oils	Treat	Cov	Cov*Treat
CGIT					
Basal glucose conc. (mg/dL)	91.0 ± 1.0	91.9 ± 1.0	0.90	0.0005	0.93
Glucose 45 min conc. (mg/dL)	101.2 ± 6.39	99.6 ± 6.39	0.46	0.0002	0.42
Delta 45 glucose conc. (mg/dL)*	9.8 ± 6.08	8.0 ± 6.08	0.90	0.0003	0.60
Insulin 75 min conc. (μIU/mL)^a^	53.9 ± 7.25	39.1 ± 7.25	0.11	<0.0001	0.0008^a^
Glucose clearance rates in positive phase (mg/dL/min)	2.5 ± 0.53	2.9 ± 0.52	0.86	0.003	0.57
Positive phase glucose AUC (mg/dL/min)	2429.9 ± 232	2148.5 ± 232	0.31	0.0006	0.15
Time in positive phase (min)^a^	72.6 ± 11.66	61.5 ± 11.66	0.14	0.0002	0.04^a^
Lowest positive phase glucose conc. (mg/dL)	93.8 ± 1.65	95.6 ± 1.65	0.92	0.002	0.96
Nadir glucose conc. (mg/dL)	74.4 ± 3.57	74.0 ± 3.57	0.29	0.003	0.28
Time until nadir (min)	113.6 ± 8.15	105.4 ± 8.15	0.22	0.007	0.16
OST					
Delta insulin (μIU/mL)*^a^	72.3 ± 8.13	47.7 ± 8.13	0.71	<0.0001	0.008
Delta glucose (mmol/L)*	2.6 ± 0.16	2.6 ± 0.16	0.35	0.27	0.34

AUC, area under the curve; Treat, treatment; Cov, covariate; CGIT, combined glucose insulin tolerance test; OST, oral sugar test. *Delta for CGIT: *t* = 45 minus *t* = 0 glucose concentrations; delta for OST: *t* = 60 minus *t* = 0 concentrations. Data are presented as least square means ± standard error of the mean.

a Data with a significant covariate by treatment interaction are presented in Figure 1.

### Feed challenge

**Figure 2:**
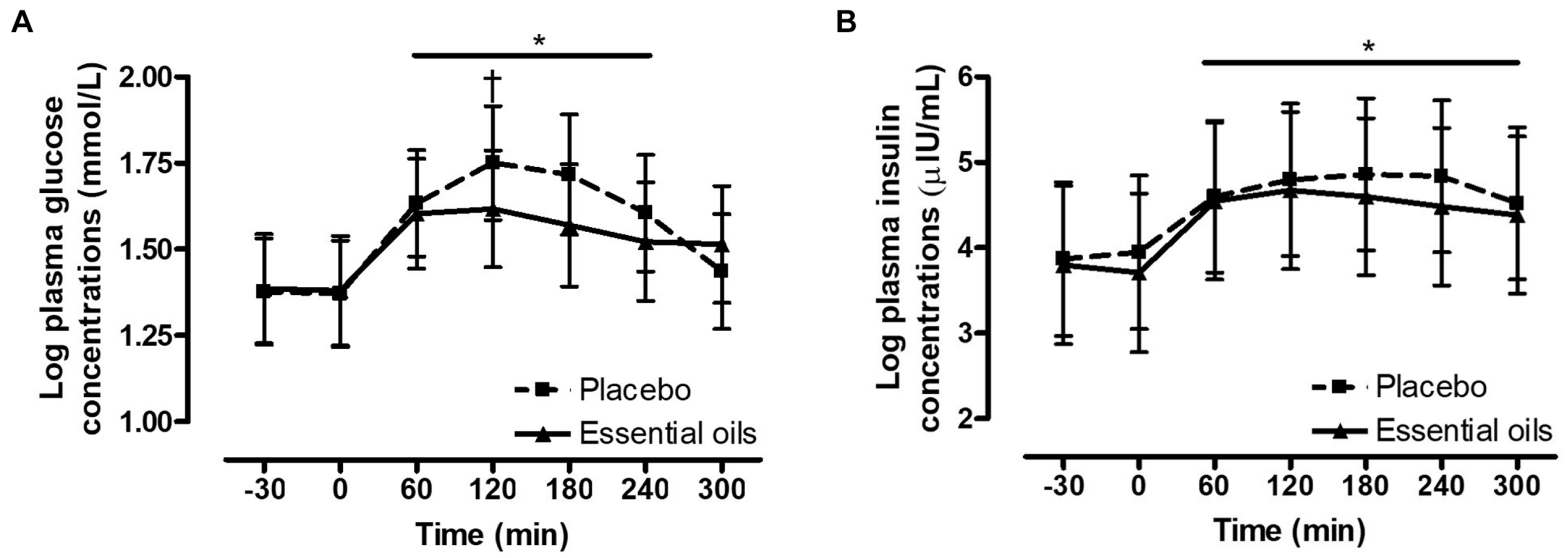
Effect of essential oils and placebo supplementation on postprandial glucose and insulin responses to feeding a meal of whole oats. Postprandial plasma log glucose and log insulin concentrations following consumption of a meal of whole oats providing 1 g starch/kg BW. **(A)**: effect of treatment on glucose concentrations (mmol/L) at baseline (−30, 0 min) and 60, 120, 180, 240 and 300 min post feeding. *Indicates differences between time points across treatments (Effect of time, *p* < 0.0001). †Indicates trend for a treatment difference within a time point (*p* = 0.1). **(B)**: effect of supplementation on log insulin concentrations (μIU/mL) at baseline (−30, 0 min) and 60, 120, 180, 240 and 300 min post feeding. *Indicates differences between time points across treatments (Effect of time, *p* = 0.002). Dotted line with squares: placebo (*n* = 10); Solid line with triangles: essential oils (*n* = 10). Data are presented as least square means ± standard error of the mean.

There was an effect of time on postprandial plasma glucose concentrations (*p* < 0.0001, Figure 2A). Glucose concentrations increased over baseline at 60 and remained elevated over basal levels for the majority of the collection period (*p* ≥ 0.006). Concentrations tended to be back to baseline by 300 min (*p* = 0.07). Additionally, there was a trend for a treatment by time interaction for the plasma glucose response post feeding a high starch meal (*p* = 0.10, Figure 2A). Plasma glucose concentrations were lower (*p* = 0.10) in the EO group (9.12 mmol/L (95% CI: 8.10–10.27)) at 120 min compared to the placebo (9.66 mmol/L (95% CI: 8.50–10.98)) group. There was no difference in postprandial glucose concentrations at any of the other timepoints (*p* ≥ 0.19, Figure 2A). As expected, there was an effect of time on postprandial plasma insulin concentrations (*p* = 0.002, Figure 2B) where insulin increased over baseline levels at 60 min and remained significantly elevated over basal concentrations for the entire duration of the collection period (*p* ≥ 0.01). There was no effect of treatment on the plasma insulin response post feeding the high starch meal (*p* = 0.3, Figure 2B).

### Oral sugar test

Six weeks of essential oil treatment reduced the insulin response to the OST (i.e., delta between 60 and 0 min) in horses with severe initial hyperinsulinemia (treatment*covariate *p* = 0.008). Delta insulin concentrations were reduced after 6 weeks of EO treatment for ID horses with “pre” insulin concentrations ≥62 μIU/mL. Horses with an average delta insulin concentration of 62.0 μIU/mL at the start of the study (i.e., the average level of the covariate) had plasma insulin concentrations of 70.5 ± 6.27 and 46.9 ± 6.27 μIU/mL after supplementation in the placebo and EO group, respectively (*p* = 0.006, Figure 1C, Table 3). Horses with an average delta insulin concentration of 179.0 μIU/mL at the start of the study (i.e., the maximum level of the covariate) had plasma insulin concentrations of 155.0 ± 12.55 and 78.1 ± 14.76 μIU/mL after supplementation in the placebo and EO group, respectively (*p* = 0.001, Figure 1C). EO supplementation did not affect the glucose response to the OST (*p* = 0.35, Table 3).

### Metabolomics

**Figure 3:**
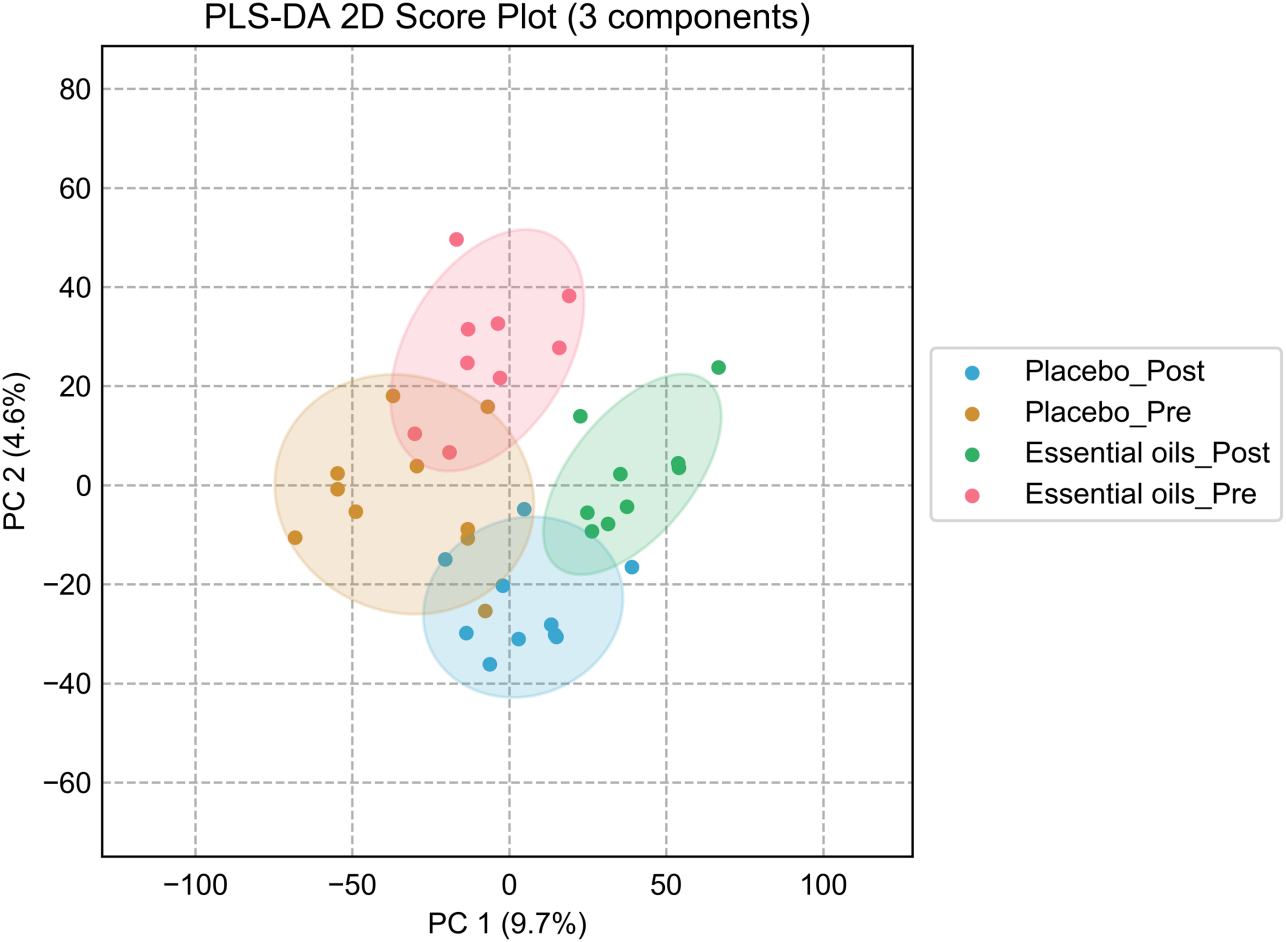
Partial least squares discriminant analysis plots. Score plot of the PLS-DA analysis for the placebo_pre (brown, placebo prior to onset of the study), placebo_post (blue, placebo after 6 weeks of treatment), essential oil_pre (red, essential oil group prior to onset of the study), essential oil_post (green, essential oil group after 6 weeks of treatment). Permutation test was carried out for 100 times with empirical *p*-value <0.01.

Four-channel metabolomic analysis revealed a total of 10,281 metabolites (Supplementary Table 1) in the plasma samples belonging to 4 submetabolomes: carboxyl, amine/phenol, carbonyl and hydroxyl. Amongst these metabolites, 566 were detected in Tier 1 (positively identified metabolites), 1939 in Tier 2 (high-confidence putatively identified metabolites), and 6,312 in Tier 3 (putatively identified metabolites). Partial least squares discriminant analysis (PLS-DA) was performed to determine the effects of both time and treatment on the plasma metabolome. While there was some overlap, there was noticeable clustering of plasma metabolites according to time and treatment (i.e., essential oil _pre, essential oil _post, placebo_pre, placebo_post, Figure 3). After 6 weeks of supplementation, there was clear separation between the 2 treatment groups, with the essential oil group showing the largest degree of separation with component 1, indicating the supplement caused a unique shift in the plasma metabolome. The permutation test result can be found in the Supplementary Figure 1. However, it is evident there was time-wise separation in both treatment groups.

**Figure 4:**
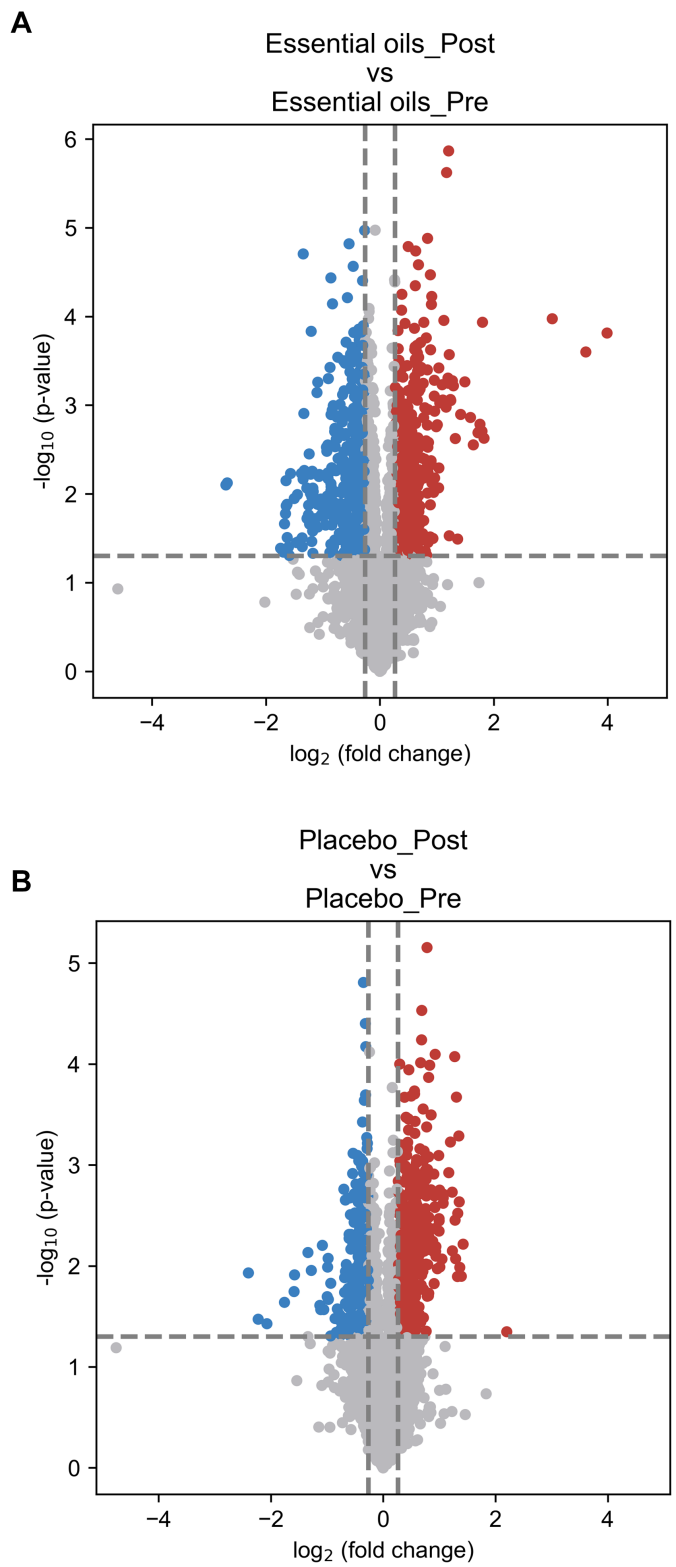
Volcano plots illustrating changes over time. Paired volcano plot for **(A)**: essential oil treated horses over time and **(B)**: placebo treated horses over time. The fold change (FC) was calculated as Mean (treatment_Post)/Mean (treatment_Post). *p*-value was calculated using paired t-test. FC > 1.2 or < 0.83 and *p* < 0.05 was used as the criteria to determine the significant metabolites. In panel **(A)** comparisons of the essential oil treatment groups, 611 upregulated (red) and 578 downregulated (blue) metabolites were found. In panel **(B)** comparisons of the placebo treatment groups, 542 upregulated (red) and 318 downregulated (blue) metabolites were found.

Paired volcano plot as the univariate analysis showed that 1,189 metabolites changed over time (611 upregulated and 578 downregulated) in the essential oil group (*p* < 0.05, Figure 4A) and 860 metabolites (542 upregulated and 318 downregulated) in the placebo group (*p* < 0.05, Figure 4B). The significances of all metabolites were presented in the Supplementary Table 3. The false-discovery rate adjusted *p*-value (i.e., *q*-value) was calculated and shown in the Supplementary Table 3. In this study, we did not use *q*-value to determine the significantly changed metabolites, to avoid missing any potential valuable information for this discovery study. In addition, there were 388 overlapping metabolites that were changed over time in both treatment groups. In order to isolate the effect of essential oils on the plasma metabolome, these overlapping metabolites were subsequently removed for further analysis.

**Figure 5:**
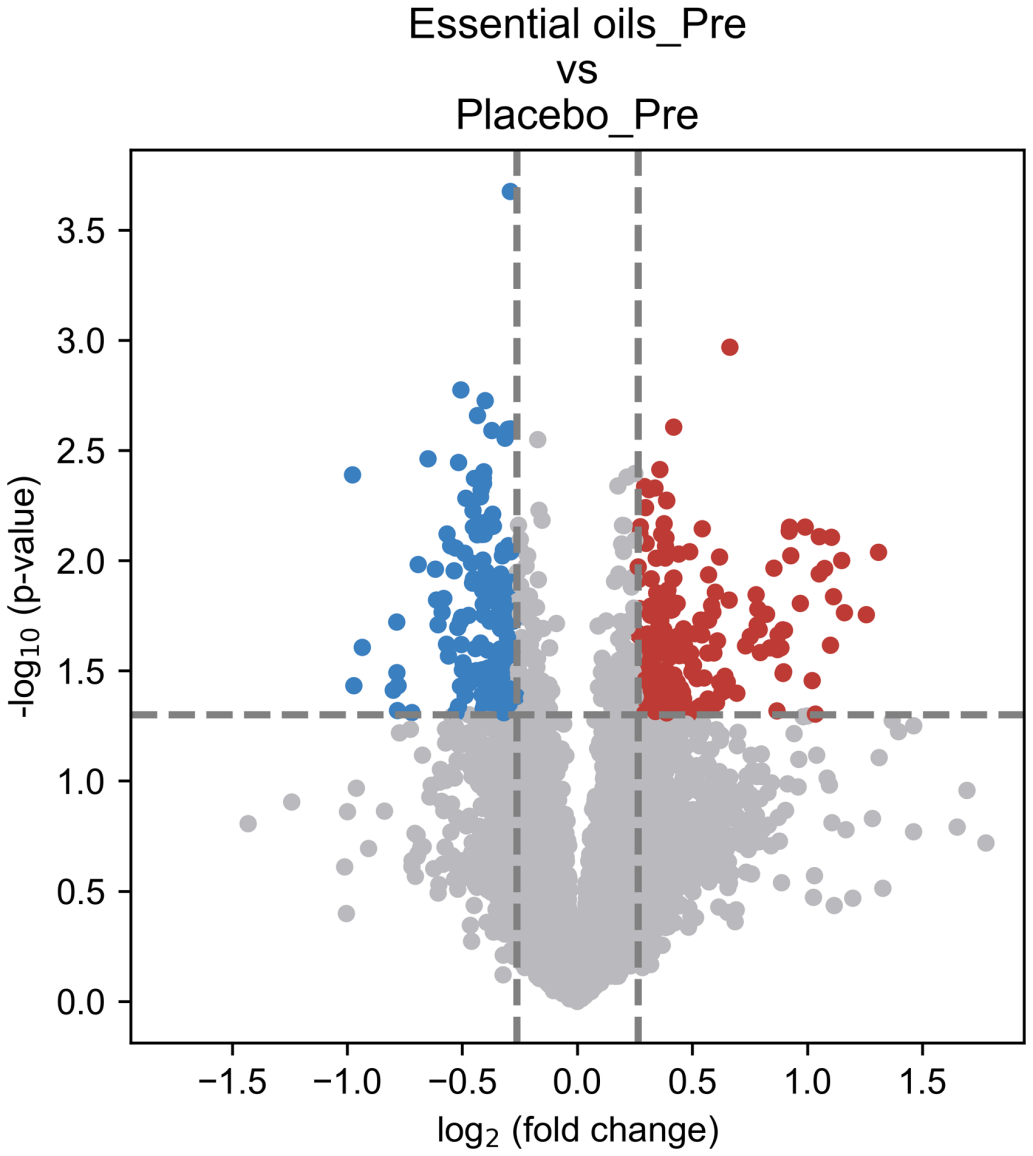
Volcano plot illustrating treatment differences prior to onset of the study. Univariate analysis for placebo and essential oil treated horses before start of the study. The fold change (FC) was calculated as Mean (Essential oils_Pre)/Mean (Placebo_Pre). *p*-value was calculated using t-test. FC > 1.2 or < 0.83 and *p* < 0.05 was used as the criteria to determine the significant metabolites [216 upregulated (red) and 181 downregulated (blue)].

**Figure 6:**
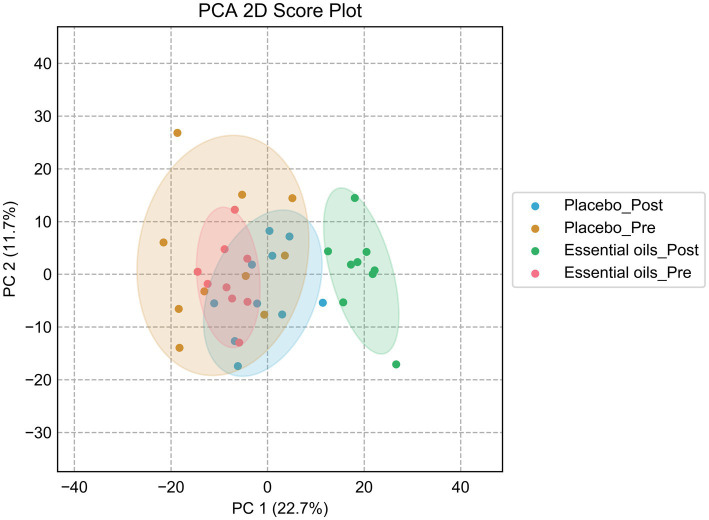
Principal component analysis plot after sequential removal of the overlapping metabolites. PCA score plot for the placebo_pre (brown, placebo prior to onset of the study), placebo_post (blue, placebo after 6 weeks of treatment), essential oil_pre (red, essential oil group prior to onset of the study), essential oil_post (green, essential oil group after 6 weeks of treatment).

**Figure 7:**
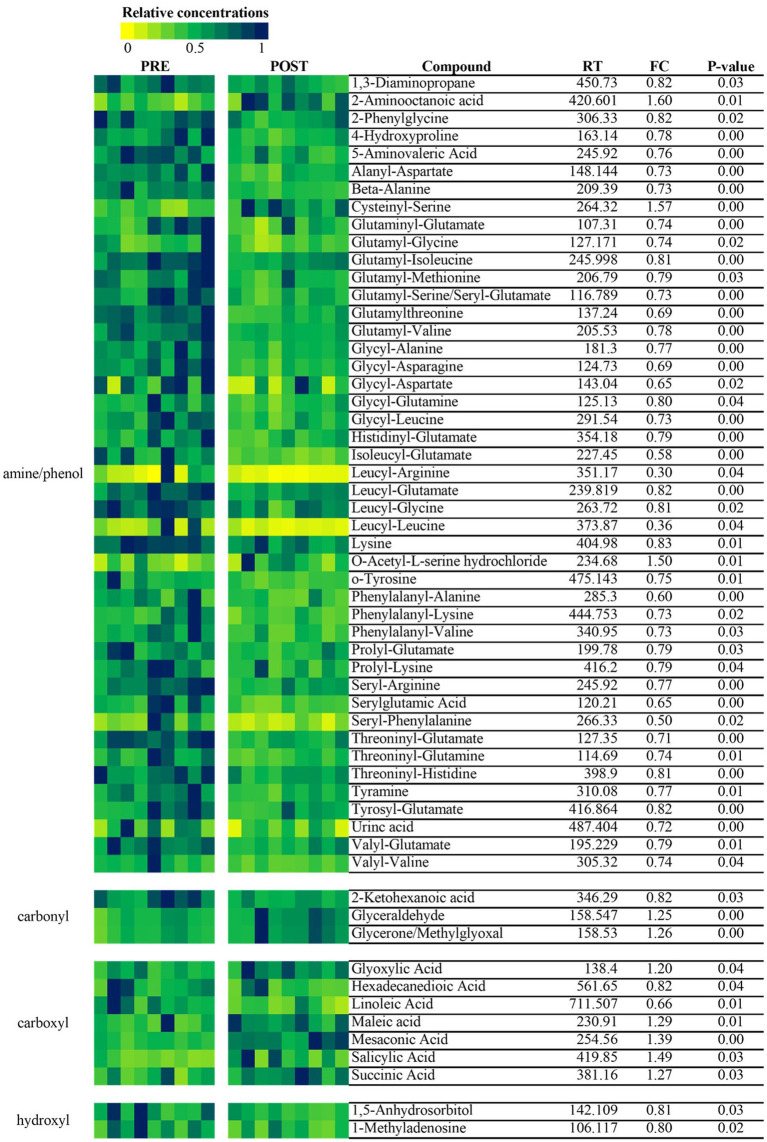
Heatmap of metabolites that were significantly changed over time within the essential oil treated horses. Essential oil-specific metabolic panel before and after 6 weeks of supplementation. The heatmap was created using the 57 positively identified biomarker metabolites (Tier 1) in the essential oil group. Data were normalized to the maximum value per compound.

Despite similar housing and feeding conditions and treatment groups being balanced for insulin status, age and body weight, univariate analysis also identified 397 metabolites that were different between the treatment groups at the start of the study (Figure 5). Of these, 55 were overlapping metabolites that also changed over time in the essential oil group and thus were removed from the dataset, leaving 746 metabolites that were uniquely changed over time with essential oil supplementation. Principal component analysis (PCA) of this dataset revealed clear separation with component 1 of the essential oil group from the other 3 groups, the latter of which showed a high degree of overlap (Figure 6). Of these 746 metabolites, 67 were identified in Tier 1, 151 in Tier 2, 446 in Tier 3 and 82 were unidentified. Next, all plant-related compounds were removed from the data set, leaving a final of 57 treatment-related biomarkers identified in Tier 1 and 117 in Tier 2 (Figure 7 and Supplementary Table 2) that were uniquely changed with essential oil supplementation. Most of the positively identified Tier 1 metabolites were dipeptides that showed a general decrease (*p* ≤ 0.04, FC ≥ 0.3) with 6 weeks of essential oil supplementation. (Figure 7). However, a few metabolites with the largest fold change over time were increased with essential oil supplementation (Supplementary Table 2): 2-Aminooctanoic acid (1.6 fold increase, *p* = 0.007), Cysteinyl-Serine (1.6 fold increase, *p* = 0.0006), O-Acetylserine (1.5 fold increase, *p* = 0.01), Mesaconic acid (1.4 fold increase, *p* = 0.003), Succinic acid (1.3 fold increase, *p* = 0.03), Glyceraldehyde (1.2 fold increase) and Glyoxylic Acid (1.2 fold increase, *p* = 0.04).

**Figure 8:**
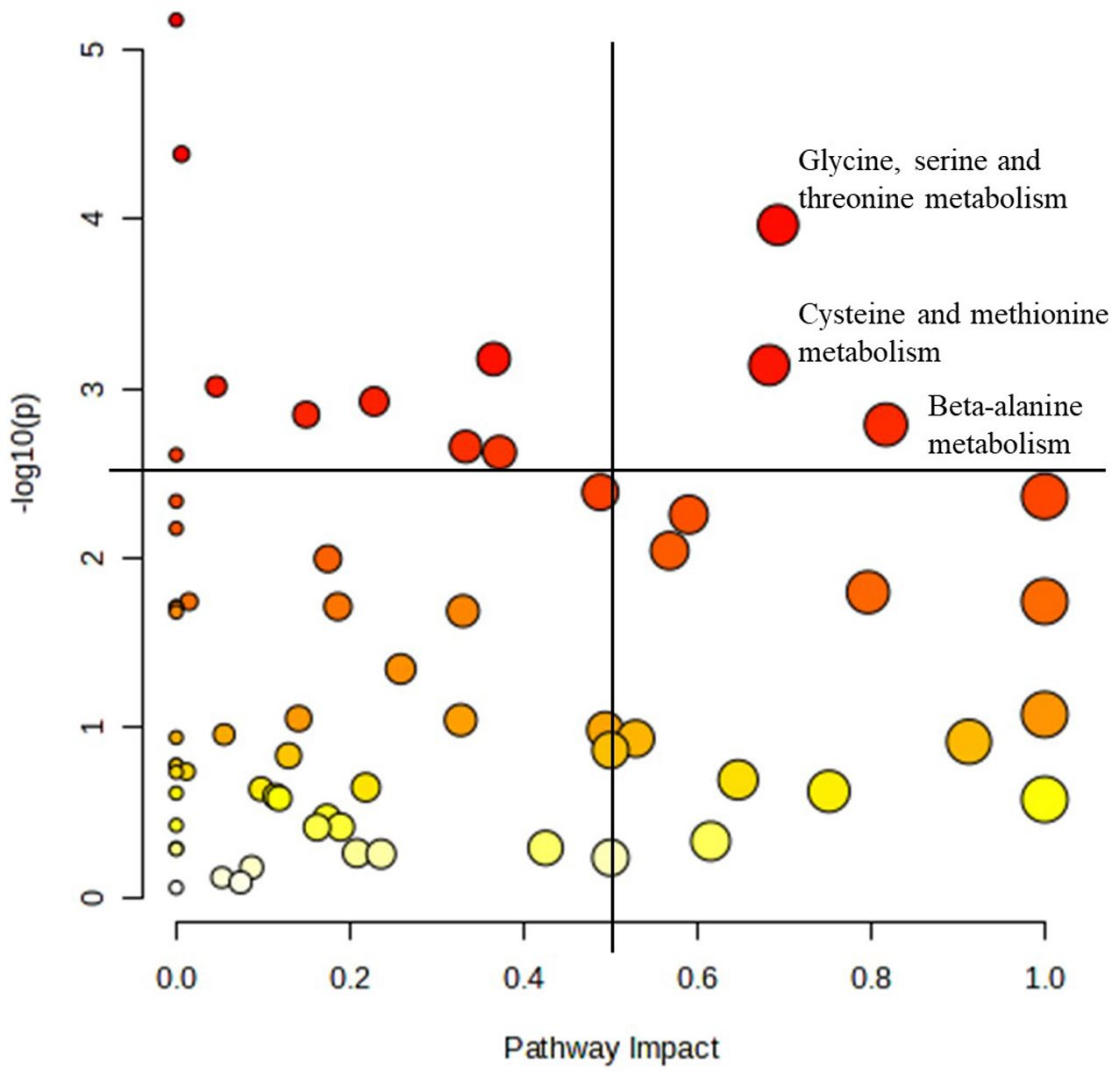
Pathways most significantly impacted in the essential oil treated horses. Pathway analysis of the plasma metabolome before and after 6 weeks of essential oil supplementation. Pathways with an impact factor > 0.5 and a -log10(p) > 2.5 were identified as most significantly impacted. All other significant pathways are listed in Table 4.

For the reasons described above, pathway analysis focused on metabolome changes of horses receiving the essential oil treatment (i.e., pre_ essential oil vs. post_ essential oil). For this analysis all 1,215 metabolites identified in Tier 1 and 2 were included. Although a number of pathways were shown to be impacted by essential oil supplementation (Table 4), we focused on those with a -log10(p) > 2.5 and an impact factor > 0.5. This resulted in identification of 3 pathways (Figure 8) that were significantly altered in the essential oil group over time: glycine, serine, threonine metabolism, cysteine and methionine metabolism and beta-alanine metabolism (Supplementary Figures 2–4). The metabolites significantly affected in the glycine, serine, threonine pathways (Supplementary Figure 2) were L-threonine (*p* = 0.02), 2-Oxobutanoate (*p* = 0.006), L – 2 Amino-3-oxobutanoic acid (*p* = 0.0004), Aminoacetone (*p* = 0.004), L-Cysteine (*p* = 0.005), Pyruvate (*p* = 0.03) and Glyoxylate (*p* = 0.05). Within the cysteine, methionine metabolism pathway (Supplementary Figure 3) there was a significant change in L-Cysteine (*p* = 0.005), L- Cystine (*p* = 0.001), Thiocysteine (*p* = 0.02), Pyruvate (*p* = 0.03), 2-Oxobutanoate (*p* = 0.006), 3- Sulfino-L-alanine (*p* = 0.003) and S-Adenosyl-L-homocysteine (*p* = 0.07). Lastly, the following metabolites were impacted with essential oil treatment in the beta-alanine metabolism pathway (Supplementary Figure 4): 1,3 Diaminopropane (*p* = 0.01), 3 – Aminopropanal (*p* = 0.06), Beta-alanine (*p* = 0.0002), Malonate semialdehyde (*p* = 0.06) and Aspartate (*p* = 0.02). Separate pathway analysis was conducted for those horses with most severe initial degree of insulin resistance and responded most strongly to the essential oil treatment (*n* = 4). All significantly altered pathways and the directional change for their metabolites for this group overlapped with those for the complete essential oil cohort, with the exception of primary bile acid biosynthesis (*p* = 0.01, data not shown). In this pathway, glycocholine, a conjugated form of cholate, was increased with essential oil treatment (*p* = 0.01).

**Table 4:** Metabolic pathways significantly impacted by 6 weeks of essential oil supplementation.

Pathway name	Match status	*p*	-log(p)	Holm p	FDR	Impact
Biosynthesis of unsaturated fatty acids	5–36	6.77E-06	5.17	4.40E-04	4.40E-04	0
Lipoic acid metabolism	4–28	4.17E-05	4.38	0.003	0.0014	0.01
Glycine, serine and threonine metabolism	17–34	1.09E-04	3.96	0.007	0.0024	0.69
Arginine biosynthesis	6–14	6.71E-04	3.17	0.042	0.0095	0.37
Cysteine and methionine metabolism	18–33	7.30E-04	3.14	0.045	0.0095	0.68
Propanoate metabolism	7–22	9.74E-04	3.01	0.058	0.0106	0.05
Citrate cycle (TCA cycle)	5–20	1.19E-03	2.92	0.070	0.0111	0.23
Pantothenate and CoA biosynthesis	11–20	1.43E-03	2.85	0.083	0.0116	0.15
β-Alanine metabolism	11–21	1.64E-03	2.79	0.093	0.0118	0.82
alpha-Linolenic acid metabolism	2–13	2.20E-03	2.66	0.123	0.0133	0.33
Lysine degradation	9–30	2.38E-03	2.62	0.131	0.0133	0.37
Valine, leucine and isoleucine biosynthesis	7–8	2.46E-03	2.61	0.133	0.0133	0.00
Arachidonic acid metabolism	17–44	4.09E-03	2.39	0.217	0.0200	0.49
Linoleic acid metabolism	3–5	4.32E-03	2.36	0.225	0.0200	1.00
Thiamine metabolism	1–7	4.62E-03	2.34	0.236	0.0200	0.00
Histidine metabolism	10–16	5.53E-03	2.26	0.277	0.0225	0.59
Biotin metabolism	1–10	6.68E-03	2.18	0.327	0.0255	0.00
Alanine, aspartate and glutamate metabolism	14–28	9.03E-03	2.04	0.433	0.0326	0.57
Butanoate metabolism	7–15	0.010	2.00	0.474	0.0345	0.17
Arginine and proline metabolism	22–36	0.016	1.80	0.730	0.0499	0.80
Taurine and hypotaurine metabolism	5–8	0.018	1.75	0.808	0.0499	1.00
Fatty acid biosynthesis	4–47	0.018	1.74	0.808	0.0499	0.01
Glutathione metabolism	7–28	0.019	1.72	0.829	0.0499	0.19
One carbon pool by folate	1–9	0.019	1.71	0.829	0.0499	0.00
Ubiquinone and other terpenoid-quinone biosynthesis	4–18	0.020	1.71	0.829	0.0499	0.00
Glycerolipid metabolism	3–16	0.020	1.69	0.829	0.0499	0.33
Retinol metabolism	1–17	0.021	1.68	0.829	0.0499	0.00
Pyrimidine metabolism	10–39	0.045	1.35	1.000	0.1043	0.26

## Discussion

Essential oils have been used in traditional medicine for centuries. However, the increasing scientific evidence of their therapeutic benefits as well as the trend for use of natural and green products in management of human and veterinary health and disease, paved the way for more extensive research and use of essential oils in the last decade.

Essential oils are volatile and concentrated extracts derived from aromatic plants, containing a wide variety of bioactive compounds such as terpenes, phenolics, and aliphatic molecules (25). These natural compounds exhibit diverse pharmacological properties, including antimicrobial, anti-inflammatory, analgesic, and antioxidant effects (26). In clinical practice, essential oils are applied through various methods, including aromatherapy, topical application, and ingestion, depending on the specific medical condition and the oil’s properties. Numerous studies have also explored the use of essential oils for conditions such as chronic pain management (27), mood disorders (28), neurologic diseases (29) and as even curative treatments for infectious diseases (30), cancer (31) and metabolic diseases such as diabetes (32) in human and rodent models.

Essential oil related research in horses is limited though an increasing number of publications looking at potential health benefits have emerged in recent years. Several studies have evaluated the use of lavender essential oil as a stress modulator in horses (33, 34). One study showed that topical application of lavender essential oil on the nostrils mitigated elevation in heart rates and salivary cortisol levels compared to control horses during various stress tests (13). An *in vitro* study using essential oils of thyme and oregano showed the potential for these oils to control *Staphylococus xylosus* infections (35). Oral administration of essential oils containing carvacrol, thymol, guaiacol, m-cresol and eugenol essential oil for 20 days did not affect apparent nutrient digestibility, fecal pH, microbial populations or postprandial plasma glucose and insulin responses (15, 36). A recent study looked at the effect of thyme essential oil on the serum biochemical profile in endurance horses and observed a decrease in total cholesterol, uric acid, alanine amino transferase, aspartate aminotransferase, triglycerides, creatinine and urea in supplemented horses (14). Our current study extends these findings to include beneficial effects on insulin and glucose metabolism. Albeit more work on chemical composition and dosage is needed in horses, these data suggest essential oils have potential as functional ingredients to modulate health and performance of horses.

The pharmaceutical and therapeutic properties of essential oil and their mode of action are dependent on their chemical composition, which varies from plant to plant. The constituents of essential oils are typically categorized as either volatile (including monoterpenes, sesquiterpenes, oxygenated derivatives, alcohols, aliphatic aldehydes and esters) or non-volatiles fractions (including carotenoids, fatty acids, flavonoids and waxes) (37). Each of these have distinct properties, for example hydocarbons are shown to elicit antimicrobial activity, certain terpenes have anti-inflammatory, anti-fungal, antimicrobial and anticancer activity, aldehydes have antifungal, circulatory, anti-inflammatory and cardiovascular benefits, phenols have antibacterial functions and immune-modulating effects, certain ketones has shown to have anti-viral, gastrointestinal regenerative actions and analgesic effects, etc. (25). The supplement used for the current study comprised of a blend of 12 different essential oils. Although the specific formula was proprietary, it included a wide variety of essential oils, including *Eucalyptus globulus*, *Allium sativum* and *Betula alba*, of which the chemical composition and mode of action has been previously described.

After 6 weeks of supplementation with essential oils, time in the positive phase during the CGIT was reduced, indicating that glucose was cleared faster from the blood. Furthermore, plasma insulin concentrations at 75 min during the CGIT were lower in the essential oil supplemented horses compared to horses receiving the placebo treatment. Time in positive phase and insulin concentrations at 75 min are two standard parameters used to assess peripheral insulin sensitivity during this dynamic intravenous test (20). These results indicate that 6 weeks of essential oil supplementation improved peripheral insulin sensitivity. When challenged with an oral starch load, there was a trend for plasma glucose concentrations to be lower in the essential oil treated horses compared to the placebo treatment. Considering postprandial plasma insulin levels were similar between the groups, this suggests that glucose tended to be cleared faster from the blood due to increased tissue insulin sensitivity. When the ID horses were administered corn syrup, animals supplemented with essential oils had a significantly lower insulin response compared to the placebo group. In contrast to the starch load, the glucose response was not different between the groups, indicating that at similar glucose loads, hyperinsulinemia seemed to be mitigated after 6 weeks of essential oils administration.

Interestingly, the effects of essential oil supplementation were most evident in horses with greater degree of ID at the start of the experiment. In order to account for inter-horse variation, all data was analyzed using each individual horse’s baseline measurement as a covariate and treatment effects were evaluated at different levels of the covariate. This unique analysis enabled us to assess the impact of supplementation across a range of severity of insulin dysregulation. As expected, treatment intervention was statistically more significant in animals suffering from more severe metabolic disruption compared to horses with only mild ID. This does not suggest that the essential oil treatment was only effective in horses with severe disease but rather most likely reflects the limitation of the measures in detecting differences in horses at the lower end of the insulin resistance spectrum. This is supported by the shifts in the plasma metabolome between the entire cohort of the essential oil horses versus those with most severe disease.

The insulin-sensitizing effect of certain essential oils has been previously described. An *in vitro* study elegantly evaluated the anti-diabetic effects of 29 essential oils (38). Several oils including essential oils of lemon balm, peppermint, lavender, bergamot, cypress, niaouli nerolidol, geranium-rose and revensara were able to improve cellular glucose utilization and inhibited lipid accumulation (38). Consequent chemical analysis of these oils revealed that in particular citrals are crucial to their anti-diabetic effect and work through activation of the AMPK/ACC pathway (38). As a crucial fuel sensitizing enzyme, AMPK plays as essential role in energy metabolism by increasing glucose uptake and decreasing lipid accumulation. An *in vivo* study using diabetic mice observed similar results where supplementation with citral-rich essential oils resulted in improved glucose tolerance assessed by an oral glucose tolerance test (12). Further gene expression analysis illustrated that the essential oils up-regulated GLUT-4 in the liver and adipocytes as well as hepatic glucokinase and down-regulated hepatic glucose-6-phosphatase and phosphoenolpyruvate carboxykinase, indicating enhanced glucose uptake and metabolism in liver and adipose tissue and inhibition of gluconeogenesis in the liver (12). Many of the oils included in the supplement of the current study are rich in citrals, which may have contributed to the observed insulin-sensitizing effects.

Metabolomic profiling revealed that 6 weeks of essential oil treatment induced significant changes in the plasma metabolome. The PLS-DA score plot illustrated clear separation between the essential oil treated horses and the placebo group. Treatment with the essential oil supplement resulted in upregulation of 611 metabolites and downregulation of 578 metabolites. After stepwise exclusion of overlapping metabolites that were changed due to random effects of time or inherent animal variation, 57 metabolites were identified as potential treatment biomarkers within the essential oil group. Most of these were dipeptides, which were decreased after 6 weeks of supplementation. It is well-known that changes in amino acid metabolism play a key role in the pathogenesis of insulin dysfunction and diabetes (39). This concept has also been illustrated in horses, where basal and postprandial plasma amino acid profiles are distinctly different between animals suffering from insulin dysregulation and healthy controls (22, 40). Higher levels of circulating amino acids, in particular aromatic amino acids as well as branched-chain amino acids (BCAA) and their derivatives, have been consistently associated with increased risk for diabetes and metabolic disease in humans (41, 42). Additionally, circulating levels of alanine and glutamate have also been positively associated with risk for type 2 diabetes (42). Many of the identified dipeptides that were reduced in the current study were BCAA or glutamate associated compounds. Whether these amino acids are mechanistically involved or rather a consequence of disease development, is still debated. Evidence from rodent models have illustrated that high levels of BCAA can elicit a negative feedback loop through the mTOR-SK6 pathway resulting in decreased cellular insulin signaling (43, 44). A similar hyperactivation of a downstream effector of Sk6, rps6, after a protein-rich meal has been shown in insulin dysregulated horses (40). Furthermore, it is known that certain amino acids, including alanine, arginine, cysteine, leucine, have strong insulinotropic effects and modulate pancreatic *β*-cell function (39). This effect is also seen in horses consuming a protein-rich meal, where the postprandial rise in plasma amino acid precedes a hyperinsulinemic response in insulin dysregulated horses (22, 40). It could be speculated that a decrease in circulating amino acids and dipeptides in the current study contributed to improved insulin signaling in the peripheral tissues, of which muscle is the largest, and reduction in pancreatic insulin secretion.

Alternatively, it is also known that insulin resistance is associated with increase muscle protein turnover (45). BCAA are a major component muscle protein, and it is known catabolism of these amino acids primarily occurs in the muscle (46). Furthermore, the activity of the branched-chain *α*-ketoacid dehydrogenase complex is shown to be reduced in conditions of impaired insulin function, reducing catabolism of BCAA reflected by the characteristic elevation of their circulating levels in individuals with metabolic disease (47, 48). Improved insulin sensitivity observed with essential oil supplementation could therefore have led to reduced protein breakdown and increased catabolism of BCAA to their keto-acids, reflected by lower circulating levels of dipeptide intermediates. Besides BCAA and glutamate dipeptides there were a few other amino acids that have previously been associated with the insulin resistance phenotype that were decreased in the with improved insulin status including lysine and tyrosine (42). While it is difficult to conclude whether essential oils modulated amino acid metabolism or whether improved insulin status led to increased tissue uptake or reduced protein turnover, the data indicate that there was a favorable shift the IR-associated amino acid profile.

In addition to the biomarker metabolites, many of the pathways that were most impacted after essential oil supplementation were associated with amino acid metabolism, including *β*-alanine, glycine, serine, threonine, cysteine and methionine metabolism. As stated in the results, all pathway findings were consistent between the complete essential oil cohort as well as those horses who responded most strongly to the essential oil treatment with the sole exception of bile acid metabolism which is discussed below. Within the cysteine/methionine pathway, S-adenosyl-L-homocysteine, a direct precursor to homocysteine, was decreased with improved insulin status after 6 weeks of essential oil supplementation. Elevated levels of homocysteine have been associated with insulin resistance in humans and although the underlying mechanism is not entirely clear, its effect seems to be mediated through impairment of pancreatic β-cell function (49, 50). In the current study, the magnitude of the insulin response to both the intravenous and oral glycemic challenge was reduced by 31 and 22%, respectively (data not shown), in the supplemented horses. A reduction in S-adenosyl-L-homocysteine would be consistent with improvement in *β*-cell function after 6 weeks of essential oil administration. Additionally, several components of the cysteine pathway, including cysteine itself, were upregulated in the supplemented horses. Cysteine is the rate-limiting precursor for glutathione, an important cellular antioxidant. Cysteine treatment has previously shown to have protective effects in the pancreatic β-cells by attenuating oxidative damage (51). As argued above, the observed improvement in insulin status could have been mediated in part by enhanced β-cell function with increased levels of cysteine. A derivative of cysteine catabolism, 3-sulfino-L-alanine was also upregulated. This metabolite can be shuttled into several pathways including sulfate and pyruvate production and hypotaurine/taurine synthesis. Taurine has been linked to insulin metabolism and supplementation has shown to increase glucose tolerance and insulin sensitivity in peripheral tissues (52). Considering that several of the amino acid pathways are closely intertwined, there were several overlapping metabolites from the cysteine/methionine pathway that were upregulated in the threonine/serine/glycine pathway. Interestingly threonine and its downstream catabolic metabolite, aminoacetone were downregulated. Aminoacetone has previously been reported to form highly reactive and toxic compounds including methylglyoxal that are capable of causing oxidative damage and glycation of proteins, DNA and lipids (53). Elevated methylglyoxal stress and glycation have been associated with several metabolic pathologies including pancreatic *β*-cell dysfunction, chronic inflammatory diseases, endothelial dysfunction and diabetes (54). Moreover, there is substantial evidence that methylglyoxal alters several pathways contributing to development of insulin resistance (55). Although methylglyoxal concentrations in the supplemented horses were not changed in the plasma, this does not negate there could be differences intracellularly. Therefore, we cannot discern whether the observed beneficial effect of essential oils on insulin sensitivity are mediated through threonine metabolism. Another metabolite within the threonine, glycine, serine pathway that was observed to be upregulated was glyoxylate. High levels of glyoxylate, a product of glycolate oxidation in the peroxisomes, has been detected in clinically diabetic and prediabetic mice and considered an early marker in diagnosis of diabetes (56). However, it should be noted that there is very little data on glyoxylate metabolism in higher species, as this pathway was long thought to only occur in plants, bacteria, and fungi. Additionally, the physiological relevance of changes in plasma glyoxylate levels are in horses, is currently unknown.

The most impacted pathway in the essential oil treated group was *β*-alanine metabolism. Supplementation of carnosine, and its rate-limiting precursor β-alanine, have been associated with improved glycemic control and enhanced insulin sensitivity (57). Its effect is suggested to occur through the ability of carnosine to scavenge reactive carbonyl species and activation of antioxidant signaling pathways thereby protecting cells against oxidative damage (57). Although the apparent expectation would have been to see an increase in β-alanine, we observed a decrease in circulating plasma levels of β-alanine and several of its precursors after essential oil supplementation. It should be noted that β-alanine and carnosine are primarily bioactive intracellularly and blood levels of these metabolites are typically low. Furthermore, it’s been shown that insulin stimulates β-alanine uptake in the skeletal muscle (58). Thus, improved peripheral tissues insulin sensitivity could have led to an increase in β-alanine uptake resulting in lower circulating concentrations of this metabolite and its associated precursors in the supplemented group.

Aside from the amino acid related compounds, there were a few other interesting metabolites that were changed after 6 weeks of essential oil supplementation, including a decrease in linoleic acid and an increase in mesaconic acid. Recent genome-wide association studies using single nucleotide polymorphisms reported that elevated circulating levels of linoleic acid were associated with the insulin resistant phenotype (59). Certain isomers of conjugated linoleic acid have previously been shown to induce insulin resistance in rodents and humans through mechanisms involving downregulation of certain proliferator-activated receptors, impairing cell membrane function and increasing intramuscular fat content (60). Mesaconic acid is an isomer of itaconate, both TCA-cycle-derived immunometabolites that play a crucial role in modulating macrophage activation (61). While both molecules have received a lot of attention for their potent anti-inflammatory, antioxidant and antibacterial properties (62), there is a distinct difference between both compounds. Itaconate is known to inhibit succinate dehydrogenase activity thereby disrupting the TCA cycle and cellular respiration (63). Conversely, recent work showed that this effect is not seen for mesaconic acid, making it superior target of interest as an active pharmaceutical ingredient in the treatment of auto-immune diseases (61). In the current study, plasma mesaconic acid concentrations increased with improved insulin status in the essential oil group. Although its immune modulatory effects are typically exerted within the mitochondria of the immune cells, other studies have detected mesaconic acid fluctuations in the blood (64). To date, there is no data available on the physiological relevance of fluctuation in blood concentrations of mesaconic acid, but it is well known that the pathogenesis of most metabolic diseases, including equine metabolic syndrome, are associated with oxidative stress and immunologic perturbations (65). As described above, many essential oils have immune-modulating effects, thus it could be speculated that some of their insulin-sensitizing effects were mediated through alterations in immune-related signaling pathways.

Another study in horses that previously investigated the relationship between plasma metabolome and disease status identified specific metabolites that distinguished between healthy control horses and those with insulin dysregulation (5). Several of these metabolites were also changed in the supplemented group in the current study (i.e., 9-HODE, 3-(3-hydroxyphenyl)proprionate, octadecadienoic acid), providing evidence that these metabolites are associated with changes in insulin dynamics in horses. Additionally, these authors identified lower cholate levels in insulin dysregulated horses compared to healthy controls (5). Reduction in circulating primary bile acid has been reported in humans with T2D (66). In the current study, we detected an increase in glycocholate, a glycine-conjugated form of cholate, in those horses with the most severe initial degree of insulin resistance after 6 weeks of essential oil supplementation. This indicates that improvement in insulin sensitivity was associated with an increase in circulating primary bile acids, which would be consistent with previous findings in humans and horses. Jacob et al., also reported a decrease in TCA-cycle intermediates in insulin dysregulated ponies, which is a well-established characteristic of type-II diabetic mellitus patients (5). In the current study, improved insulin sensitivity after essential oil supplementation was associated with an increase in TCA-cycle intermediates. Plasma succinate levels were increased with essential oil supplementation. This important TCA intermediate has been shown to be downregulated in diabetic patients (67). Furthermore, we observed an increase in 2-oxoglutarate (*α*-ketoglutarate), another key intermediate in the TCA cycle, associated with the lipoic acid metabolism pathway (*p* = 0.00004) that was highly impacted in the essential oil group. This metabolite is a major carbon skeleton in nitrogen-assimilatory reaction and also known to be a master regulator of vital biological processes including intestinal immunity, antioxidant defense, energy production and epigenetic modification (68). Supplementation with 2-oxoglutarate prevents diet-induced obesity and improves glucose tolerance and insulin sensitivity (69). Upregulation of key TCA-cycle metabolites seen in the current study suggests that the observed beneficial effects of essential oil supplementation might be associated with improved energy metabolism and consequently metabolic function.

In conclusion, the current study illustrates the potential for essential oil-derived compounds to modulate insulin metabolism thereby lowering the insulin response to an oral sugar challenge and improve peripheral insulin sensitivity in hyperinsulinemic horses. The insulin-sensitizing effect of essential oil supplementation was more pronounced in horses with more severe ID. Moreover, these findings show that there is a distinct metabolomic signature associated with insulin dysregulation in horses and that it is responsive to treatment. Through metabolic profiling, we observed that the insulin-sensitizing effect of essential oil supplementation might at least in part be mediated through alterations in amino acid metabolism, linoleic acid metabolism, mesaconic acid metabolism, TCA-cycle intermediate metabolism and primary bile acid metabolism.

## Data Availability

The original contributions presented in the study are included in the article/Supplementary material, further inquiries can be directed to the corresponding author.

## Footnotes

^1^ www.mycompoundid.org

^2^ www.metaboanalyst.ca
