# Supplementary material for: Essential oil supplementation improves insulin sensitivity and modulates the plasma metabolome of hyperinsulinemic horses
Source: Front Vet Sci. 2024 Dec 2;11:1444581. doi: 10.3389/fvets.2024.1444581 (PMC11648227; doi:10.3389/fvets.2024.1444581)
Supplement: Supplementary file 1 [file Data_Sheet_1.zip › Supplementary material captions.DOCX]

Supplementary Material

1. **Supplementary Tables**

**Supplemental Table 1 Overview of all detected plasma metabolites**

**Supplemental Table 2 Overview of 746 metabolites uniquely changed over time in the EO treated horses**

**Supplemental Table 3 Overview of all significantly changed metabolites in placebo and EO group over time**

1. **Supplementary Figures**

**Supplemental Figure 1 Validation of PLS-DA model of the plasma metabolome** Permutation test results derived from permutations using the original dataset with data assigned randomly to group labels in the PLS-DA model. Q^2^ values from the permuted analyses as are right of the original data with an empirical p-value < 0.01.

**Supplemental Figure 2 Schematic representation of the glycine, serine, threonine metabolism pathway** Metabolites that were significantly changed are highlighted in red (P ≤ 0.05) and those that tended to be changed in orange (P ≤ 0.1). Each of the metabolites is accompanied by a respective box plot generated in MetaboAnalyst 5.0, illustrating the directional changes for each metabolite.

**Supplemental Figure 3 Schematic representation of the cysteine and methionine metabolism pathway** Metabolites that were significantly changed are highlighted in red (P ≤ 0.05) and those that tended to be changed in orange (P ≤ 0.1). Each of the metabolites is accompanied by a respective box plot generated in MetaboAnalyst 5.0, illustrating the directional changes for each metabolite.

**Supplemental Figure 4 Schematic representation of the β-alanine metabolism pathway**Metabolites that were significantly changed are highlighted in red (P ≤ 0.05) and those that tended to be changed in orange (P ≤ 0.1). Each of the metabolites is accompanied by a respective box plot generated in MetaboAnalyst 5.0, illustrating the directional changes for each metabolite.
