# Supplementary figures and images for: Essential oil supplementation improves insulin sensitivity and modulates the plasma metabolome of hyperinsulinemic horses
Source: Front Vet Sci. 2024 Dec 2;11:1444581. doi: 10.3389/fvets.2024.1444581 (PMC11648227; doi:10.3389/fvets.2024.1444581)

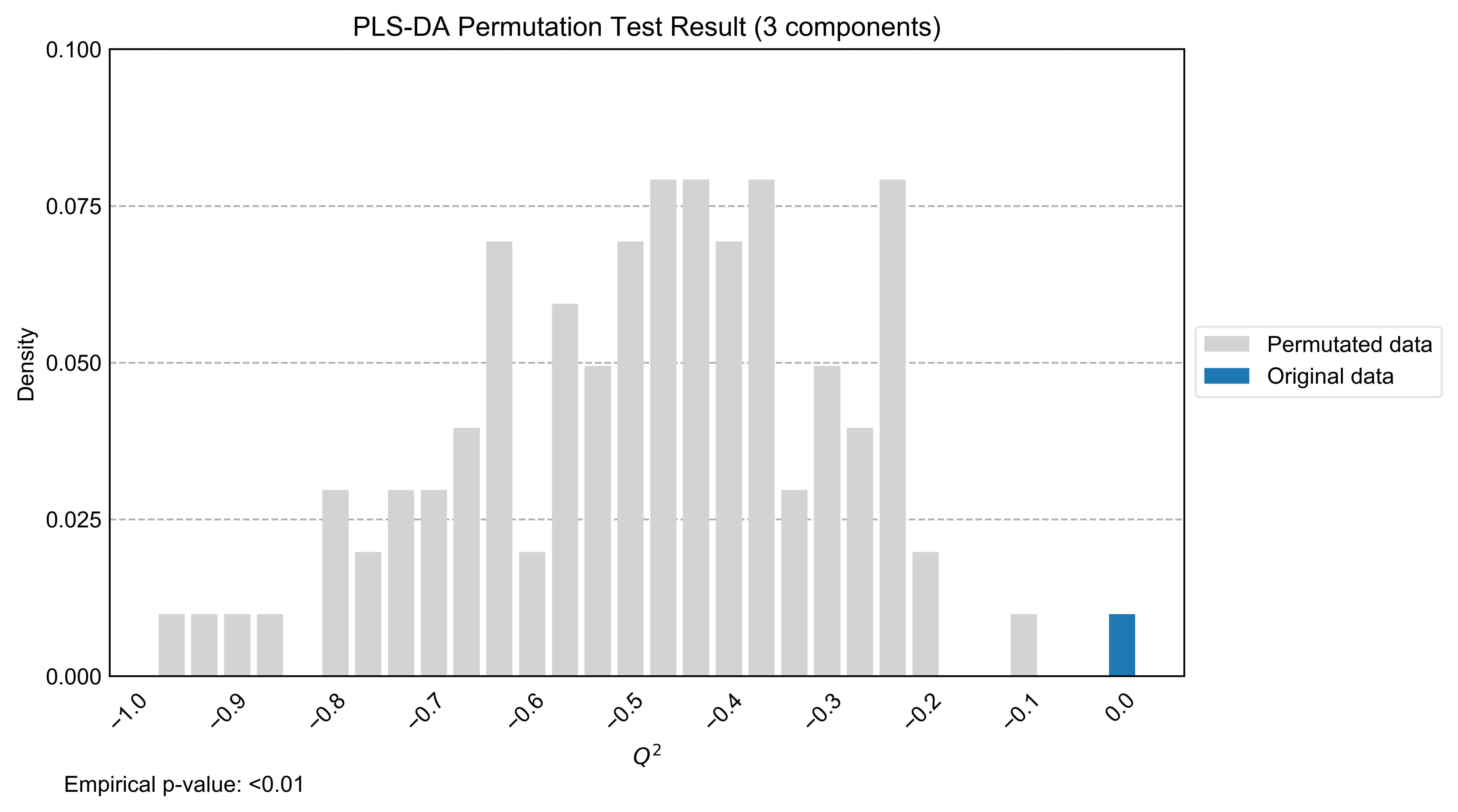

Supplement: Supplementary file 1 [file Data_Sheet_1.zip › Supplementary Figure 1.JPEG]

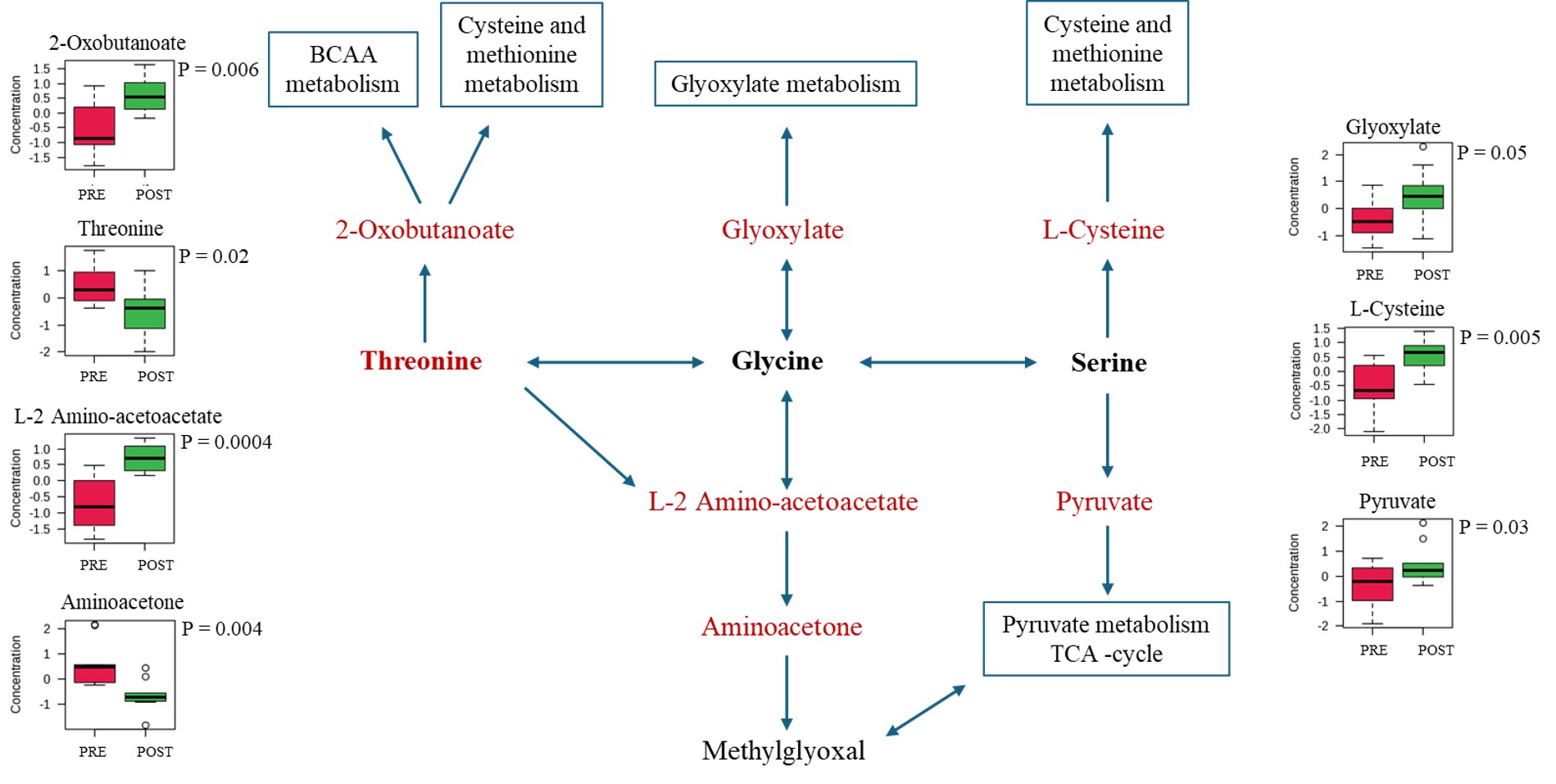

Supplement: Supplementary file 1 [file Data_Sheet_1.zip › Supplementary Figure 2.JPEG]

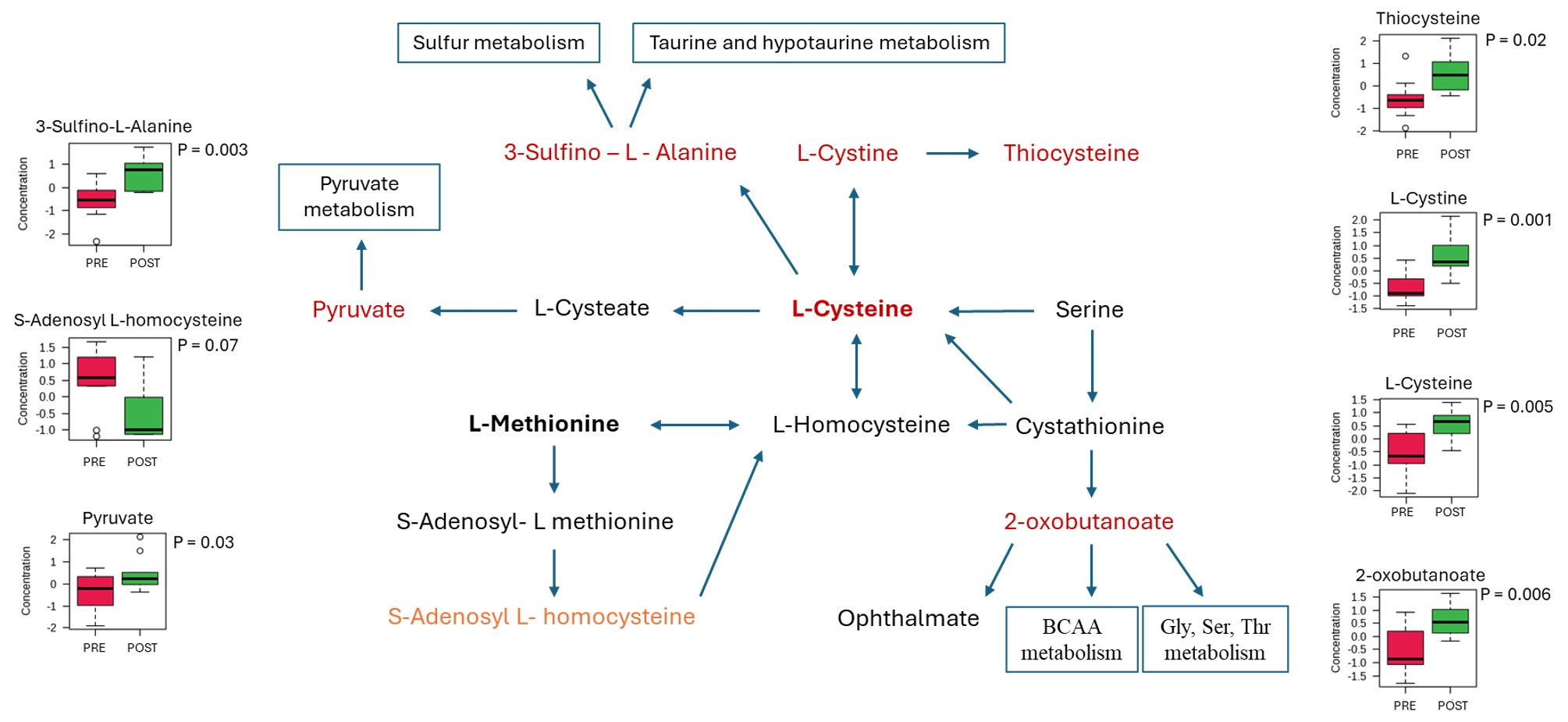

Supplement: Supplementary file 1 [file Data_Sheet_1.zip › Supplementary Figure 3.JPEG]

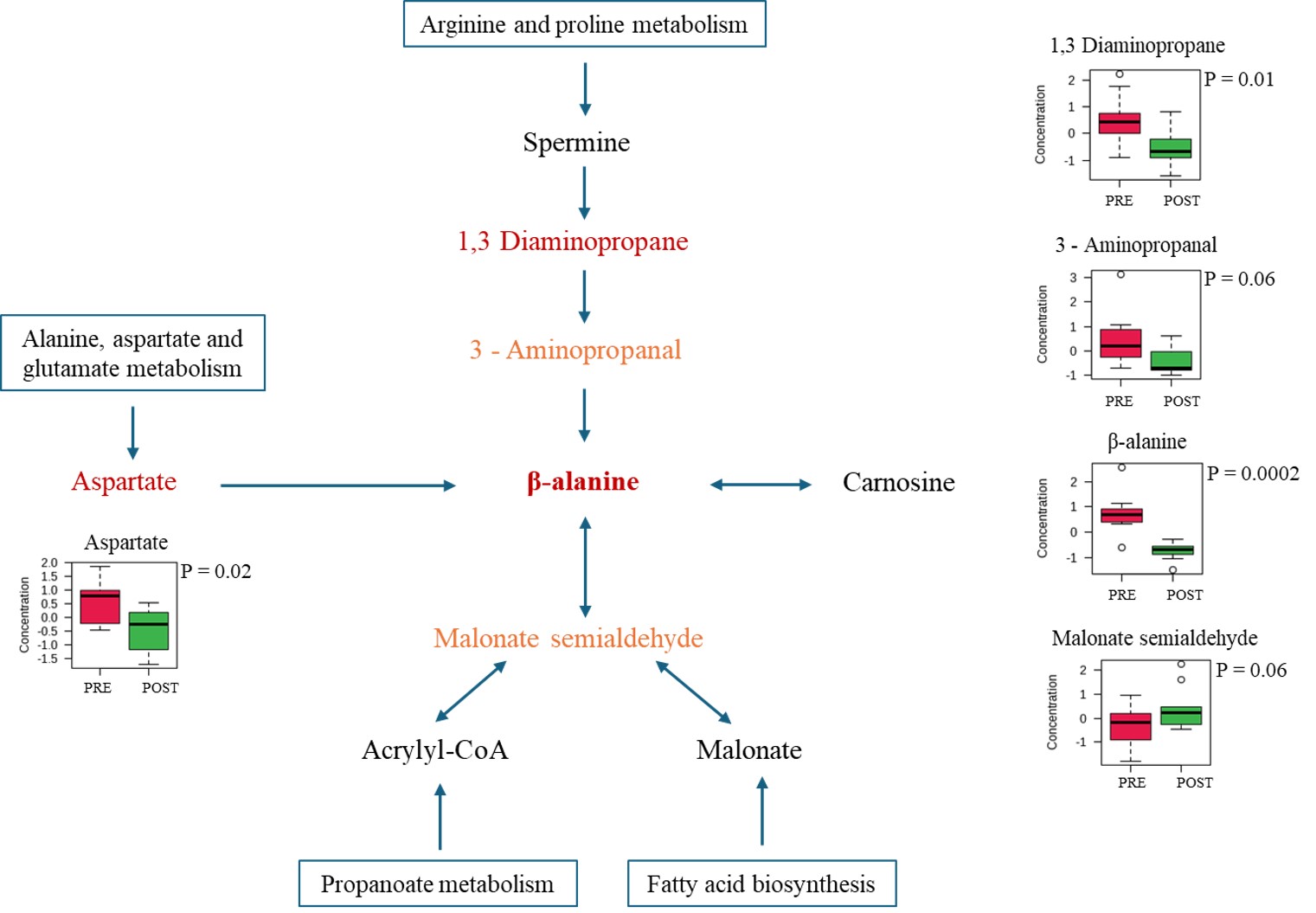

Supplement: Supplementary file 1 [file Data_Sheet_1.zip › Supplementary Figure 4.JPEG]
